# Circadian misalignment measured by social jetlag from early to late pregnancy and its association with nutritional status: a longitudinal study

**DOI:** 10.1038/s41598-021-97946-5

**Published:** 2021-09-21

**Authors:** Laura Cristina Tibiletti Balieiro, Cristiana Araújo Gontijo, Luisa Pereira Marot, Gabriela Pereira Teixeira, Walid Makin Fahmy, Claudia Roberta de Castro Moreno, Yara Cristina de Paiva Maia, Cibele Aparecida Crispim

**Affiliations:** 1 Faculty of Medicine, Federal University of Uberlândia, Uberlândia, Brazil; 2 Hospital and Municipal Maternity of Uberlândia, Uberlândia, Brazil; 3 Department of Health, Life Cycles and Society, School of Public Health, University of São Paulo, São Paulo, São Paulo Brazil; 4 Stress Research Institute, Department of Psychology, Stockholm University, Stockholm, Sweden

**Keywords:** Nutrition, Weight management

## Abstract

A mismatch between circadian and social clocks leads to a circadian misalignment, which has been widely measured by social jetlag (SJL). There are several studies measuring SJL, but it has not been studied in pregnant women. Therefore, this study aimed to identify the occurrence of SJL throughout pregnancy and to verify whether there is an effect of pre-pregnancy body mass index (BMI) on SJL throughout pregnancy. The baseline of the present study was conducted with 205 1st trimester pregnant women of whom 100 were followed in their 2nd and 3rd trimester. SJL was calculated based on the absolute difference between mid-sleep time on workdays versus work-free days. The pre-pregnancy BMI and current BMI (kg/m^2^) were calculated. Linear regression and Generalised Estimating Equation (GEE) adjusted for confounders were used to determine the association between SJL and the gestational trimesters (time), and anthropometric variables. Most of the pregnant women (54.5%) presented SJL > 1 h in the first gestational trimester. We also found an isolated effect of the gestation trimester on the SJL mean. In this sense, pregnant women had a decrease in SJL from the second to the third trimester (1.33 ± 0.08 versus 1.12 ± 0.07, respectively; *p* = 0.012). GEE analyzes showed that pregnant women of a normal weight showed a decrease in SJL from the second to the third trimester (1.29 ± 0.11 and 0.93 ± 0.08, respectively, *p* = 0.032), but this was not found in the other groups of nutritional status (underweight, overweight and obesity). In addition, a positive association between SJL and pre-gestational BMI in the third trimester (β = 0.200, *p* = 0.046) was found. SJL is quite prevalent during the gestational period and excessive BMI both before and during pregnancy is associated with an increased risk of having SJL > 1 h in the third and second trimesters, respectively. In addition, pregnant women of normal weight—but not underweight or overweight—had decreased SJL from the second to the third trimester.

## Introduction

The central and peripheral circadian clocks have the main function of synchronizing the endogenous system over a 24-h period and controlling several biological processes, such as the sleep–wake cycle^1^. Circadian misalignment occurs when endogenous circadian rhythms are not synchronized by environmental clues—or *zeitgebers*—such as light–dark cycle^2^, food intake^3–6^ and physical activity^3,7^.

Social jetlag (SJL) is a measure of the discrepancy between biological and social time resulting from a conflict between a sleep timing with and without the constraints imposed by social obligations^8^. Proposed by Wittmann and colleagues in 2006, this variable is calculated by the mean difference between the time of sleep on workdays versus work-free days^8^ and has been used as a measure of circadian misalignment. Previous studies have shown that SJL is associated with health problems, such as obesity^9,10^, metabolic disorders^10^, type 2 diabetes^11^, atherosclerotic cardiovascular disease^12–14^, as well as with higher levels of cortisol in healthy individuals^12^ and higher levels of glycated hemoglobin in patients with type 2 diabetes^15^. Recent studies conducted by our group demonstrated that SJL is associated with an increased risk of overweight, metabolic complications^16^ and a poor diet^17^ in individuals with chronic non-communicable diseases. Thus, the aforementioned evidence suggests that chronic SJL has implications for human health.

The physical and physiological changes resulting from the gestational process to support fetal development and adapt to the stress imposed on the body can significantly impact the sleep–wake pattern^18,19^, resulting in decreased hours of sleep^18,19^, poor sleep quality^19,20^, diurnal sleepiness^21^ and insomnia^22^. Moreover, pregnancy requires coordination of several physiological systems, including metabolic, endocrine, and circadian^23^, and such changes could alter biological rhythms. A recent study on this topic found that pregnancy induces changes in daily rhythms, altering both the time of onset and the amount of activity, with these changes being conserved in mice and women^24^. However, there are still many gaps in the existing knowledge about the effects of pregnancy evolution on the circadian alignment/misalignment of pregnant women. Current studies have already described negative consequences of circadian misalignment on the outcome of pregnancy in animal models^25–28^, such as the decrease in the number of live births^26,28^, prolonged the stage of labor, but not the duration of pregnancy^28^, an elevated rate of full-term pregnancy failure^25–27^ and disturbed the fetal intrauterine growth and the growth of neonatal rats^28^. However, little is known about the effect of gestational physiological time on the circadian alignment of the pregnant women.

The aim of this study was to identify the occurrence of SJL throughout the three gestational trimesters and to verify whether there is an effect of pre-pregnancy body mass index (BMI) on SJL throughout pregnancy. We hypothesized that SJL increases throughout the gestational period and that women with a higher than normal pre-gestational BMI present a higher risk of having SJL higher than 1 h throughout the pregnancy.

## Materials and methods

### Participants and ethics

This is a two phases study (baseline and longitudinal) conducted between October 2015 and February 2017 at the prenatal service of the Integrated Care Units and Clinical Hospital of the Federal University of Uberlândia, located in Uberlândia, Minas Gerais, Brazil.

Pregnant women in the first gestational trimester were invited to participate when they were waiting for prenatal consultation in the waiting room. Before the invitation, a brief explanation of the research and procedures was given. Pregnant women were recruited according to the following eligibility criteria: being older than 18 years old, not being a shift worker (including pregnant women who worked between 07:00 and 19:00 h), not using illegal substances, not being pregnant with twins, not being Human Immuno-Deficiency Virus (HIV) positive and not having diseases such as syphilis, toxoplasmosis, rubella, cytomegalovirus, varicella or having fetal malformation or anomalies in the current pregnancy, as well as those who did not provide all necessary information for the development of the study and reported using the alarm clock on weekends.

**Figure 1:**
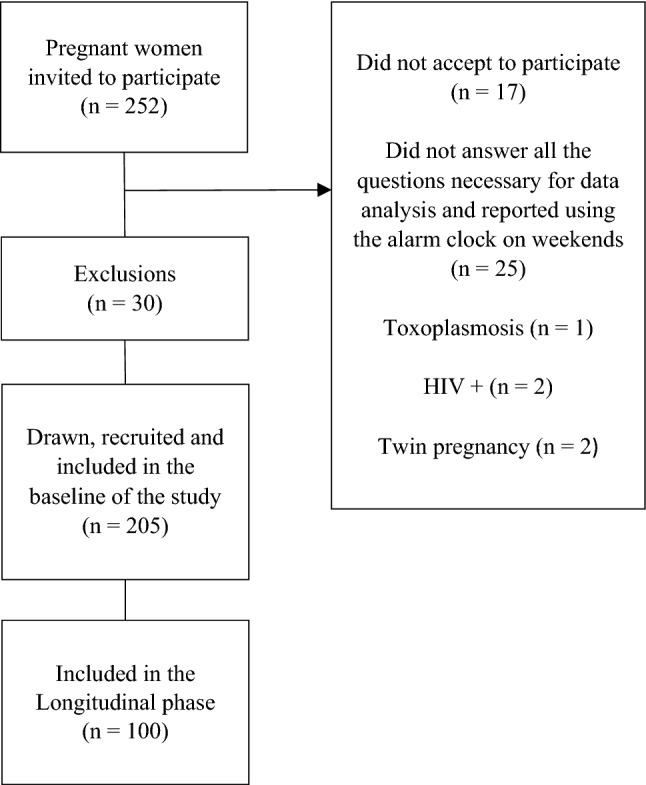
Study flowchart. *Note*: *HIV* human immuno-deficiency virus.

A total of 252 pregnant women at the first gestational trimester were invited to participate in the baseline of the study. Seventeen did not accept to participate. Thirty participants were excluded because they did not provide all necessary information (n = 25), or presented previous diseases (n = 3), or had twin pregnancy (n = 2) (Fig. 1). After the initial characterization, one hundred pregnant women were invited to participate in the second phase of the study (longitudinal phase), which involved being evaluated during the whole pregnancy during the (second and third trimester). An analysis was performed to check for possible differences between the 105 pregnant women at baseline who were not followed up and the 100 who were followed during pregnancy. These two groups do not differ for the variables age, pre-gestational BMI and SJL (*p* values obtained in the student’s t-test: *p* = 0.886, *p* = 0.518 and *p* = 0.808, respectively).

A total of 205 pregnant women were evaluated in the first trimester (4^th^ to 12^th^ gestational weeks). A questionnaire was applied by the researchers in order to evaluate socio-demographic aspects such as age, years of education, marital status and family income. Interviews and measurements were conducted by trained researchers while the pregnant women were waiting for medical appointments in the public service units.

A subsample of 100 pregnant women that were assessed in the first trimester were included in a longitudinal study and assessed over the next two gestational trimesters, evaluated between the 20^th^ and 26^th^ week and again between the 30^th^ and 37^th^ week.

Physical activity evaluation was performed by asking the pregnant if they had practiced any physical activity during the last month in each trimester (yes/no), as well as the type, frequency and duration of this physical activity. The data about morning sickness (vomiting, nausea) and heartburn was assessed by asking the pregnant if they had any episode during the last month in each trimester (yes/no), as well as the frequency. Pregnant women were asked about medication consumption (name, dosage, and frequency) and also in relation to employment status and workload. Data on physical activity, morning sickness (vomiting, nausea) and heartburn, medication use, employment status, workload and sleep pattern were self-reported in all three phases.

This study was approved by the Human Research Ethics Committee (protocol number 1.11199.829/2015) of the Federal University of Uberlândia. Research was conducted according to the guidelines in the Declaration of Helsinki. All participants signed a free and informed consent form.

### Anthropometric variables

The height and current weight were measured, and body mass index (BMI) was calculated. The current BMI was classified according to the gestational week suggested by Atalah et al. (1997)^29^ for the gestational age, as recommended by World Health Organization (WHO) (2000)^30^. Their current weight was measured over the three evaluations. The pre-pregnancy weight was self-reported and the pre-pregnancy BMI (kg/m^2^) was calculated and classified according to the WHO classification (2000)^30^. The weight gain was evaluated in each trimester by the following described steps: first, the recommended weight gain (Institute of Medicine 2009)^31^ in each trimester was calculated considering the number of gestational weeks corresponding to the interval between the evaluations, except for the first trimester, which the recommended weight gain was considered in the range of 0.5 to 2 kg per month. Then, the weight gain in each trimester was evaluated using the difference between the value of the current measured weight and the value of the previous trimester weight, or pre-gestational weight in the first trimester.

### Sleep data, social jetlag and chronotype

Pregnant women were asked to report usual bedtime, wake-up time, sleep-onset latency and usual sleep duration on weekdays and weekends during the pregnancy. The questions about this were based on the Munich Chronotype Questionnaire^2^. The questions used in the survey were “What time have you been going to sleep on weekdays?”; “How many minutes on average do you stay awake in bed before you fall asleep after lights are turned off on weekdays?”, “Did you use wake up with the help of someone or an alarm clock on weekdays?”, “What time have you been waking up on weekdays?”; “What time have you been going to sleep on weekends?”, “How many minutes on average do you stay awake in bed before you fall asleep after lights are turned off on the weekends?”, “What time have you been waking up on weekends?” and “Did you use wake up with the help of someone or an alarm clock on weekends?”. Sleep duration was computed using the weighted average of self-reported sleep duration, which considers both, weekdays and weekends, using the formula: [(Reported current weekday sleep duration × 5) + (Reported current weekend sleep duration × 2)]/7^15^.

SJL was calculated as the absolute difference between the time of mid-sleep on weekdays and weekends^8^. We classified the data into two groups: without SJL (≤ 1 h) and with SJL (> 1 h)^9^.

Chronotype was derived from the mid-sleep time on free days on weekend (MSF), with a further correction for calculated sleep debt (MSFsc)—calculated as the difference between average sleep duration on weekends and the average sleep during the week^32^.

Sleep quality was assessed by Pittsburgh Sleep Quality Index (PSQI)^33^ translated into Portuguese^34^, which has been used by other researchers in studies with Brazilian population sample^34,35^. The PSQI is an instrument widely used to measure the subjective sleep quality of sleep during the last month of pregnancy and has been validated in populations of pregnant women in previous studies^36,37^. The PSQI scores ranges from 0 to 21, with higher scores indicating worse sleep quality. Those participants who scored above a cut-off of 5 were classified as “poor sleepers”. The PSQI was applied in each trimester.

At each of the three evaluations sleep data were evaluated and SJL and chronotype were calculated.

### Statistical analysis

All statistical analyses were performed using the SPSS version 20.0 (SPSS Inc., Chicago, IL, USA), and *p* < 0.05 was considered statistically significant. Initially, the data normality was tested by Kolmogorov–Smirnov test. Categorical variables were summarized using frequencies and percentages, and continuous variables were summarized using means and standard error or median and interquartile intervals. Descriptive statistics were used to summarize participant sociodemographic, lifestyle, anthropometrics, sleep patterns and circadian data.

The Generalized Linear Models (GzLM) with linear distributions adjusted for age, marital status, schooling, work (yes or no) and gestational age (current BMI), were used to determine the association between SJL (dependent variable) and BMI categories in the baseline (n = 205) (independent variables; baseline of the study). Generalized Estimating Equation (GEE) models using linear distributions were used in the longitudinal phase to determine the effects of the gestational time [first (n = 100), second (n = 100) and third trimester (n = 100)], nutritional status and their interaction on SJL. Analyses were adjusted for age, marital status, schooling, work (yes or no), gestational age, parity, body mass index and PSQI global sleep quality score. Pairwise comparisons were performed using the Sidak sequential test in both tests (GzLM and GEE).

Linear regression analysis adjusted for confounders (age, marital status, schooling, work (yes or no), gestational age and parity) were performed to associate SJL (dependent variable) with anthropometric variables (independent) in the baseline (n = 205) and also in the analysis of the three trimesters (n = 100). Logistic regression models were used to predict the odds ratio (OR) for categories of anthropometric variables (appropriate: normal weight; inappropriate: underweight, overweight and obesity) according to having JSL > 1 h presence (> 1 h) or absence of SJL (≤ 1 h) in each gestational trimester. All analyses were adjusted for confounders and the results were expressed as the odds ratio with 95% confidence interval (CI).

## Results

Socio-demographic data, lifestyle, anthropometry, sleep patterns and chronotype data are presented in Table 1. Most women were married or lived with a partner (64.8% of all women in baseline; 79.0% of the women followed during the longitudinal phase). Regarding the pre-gestational BMI, 53.7% who participated only in the baseline and 57.0% who were followed during the longitudinal phase had normal weight at the beginning of pregnancy.

**Table 1:** Socio-demographic data, lifestyle, anthropometry, sleep patterns and chronotype of all pregnant women in the baseline and the women followed in the longitudinal phase.

Variables	Baseline	Longitudinal phase		
	1st trimester (n = 205) Mean ± SD or median [interquartile range] or n (%)	1st trimester (n = 100) Mean ± SD or median [interquartile range] or n (%)	2nd trimester (n = 100) Mean ± SD or median [interquartile range] or n (%)	3rd trimester (n = 100) Mean ± SD or median [interquartile range] or n (%)
Age, (years)	27.66 ± 9.79	27.70 ± 5.61		
Gestational age, weeks	9.79 ± 2.45	10.12 ± 2.40	24.00 ± 3.12	34.14 ± 2.61
Work (yes)	102 (49.7%)	56 (56.0%)	44 (44.0%)	43 (43.0%)
**Physical activity (no)**				
Participated in physical activity	17 (8.2%)	17 (17.0%)	21 (21.0%)	20 (20.0%)
**Marital status**				
Married or live with a partner	133 (64.8%)	79 (79.0%)		
Single	71 (35.2%)	21 (21.0%)		
**Schooling**				
Basic education complete/not complete	20 (9.8%)	5 (5.0%)		
High school education complete/not complete	159 (77.5%)	68 (68.0%)		
Higher education complete/not complete	26 (12.7%)	27 (27.0%)		
**Anthropometric variables:**				
Height (m)	1.65 ± 0.08	1.64 ± 0.06		
Pre-pregnancy weight (kg)	65.77 ± 14.32	65.49 ± 12.83		
Pre-gestational BMI (kg/m^2^)	24.46 ± 4.95	24.25 ± 4.30		
Underweight	18 (8.8%)	6 (6.0%)		
Normal weight	110 (53.7%)	57 (57.0%)		
Overweight	42 (20.4%)	24 (24.0%)		
Obesity	35 (17.1%)	13 (13.0%)		
Weight—current (kg)	67.57 ± 17.28	66.90 ± 13.44	72.02 ± 13.35	78.34 ± 13.52
BMI—current (kg/m^2^)	25.07 ± 5.19	24.80 ± 4.51	26.65 ± 4.47	28.98 ± 4.40
Underweight	20 (9.5%)	13 (13.0%)	13 (13.0%)	11 (11.0%)
Normal weight	108 (52.7%)	46 (46.0%)	42 (42.0%)	36 (36.0%)
Overweight	50 (24.5%)	27 (27.0%)	29 (29.0%)	32 (32.0%)
Obesity	27 (13.3%)	14 (14.0%)	16 (16.0%)	21 (21.0%)
**Sleep patterns and chronotype**				
Mean week sleep duration (h)	8.67 ± 1.76	8.71 ± 1.53	8.58 ± 1.56	8.41 ± 1.43
Mean weekend sleep duration (h)	9.17 ± 1.58	8.93 ± 1.33	9.02 ± 1.55	8.80 ± 1.30
Chronotype (MSFsc) (h:min)	4:16 ± 1:21	4:08 ± 1:23	4:13 ± 1:17	4:18 ± 1:12

*BMI* body mass index; *MSFsc* midsleep phase on free days corrected for sleep debt on work days. Values are presented as mean and SD (standard deviation) for normally distributed data or as median [interquartile range] for not normal distributed data or n (%).

**Figure 2:**
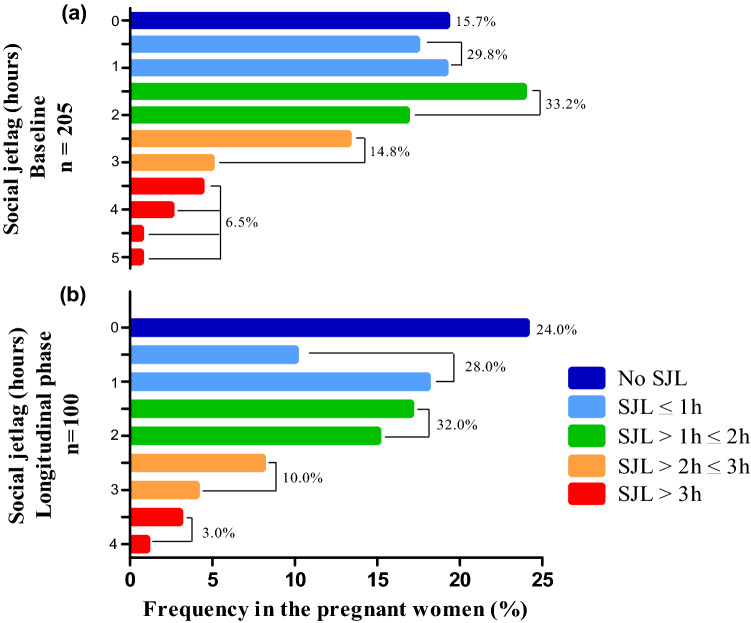
(**a**) Distribution﻿ of social jetlag (SJL) of all pregnant women in the baseline (n = 205). (**b**) Distribution of social jetlag (SJL) of all pregnant women in the longitudinal phase (n = 100) in the first trimester. *Note*: The distribution is based on half-hourly bins. Color-coding is arbitrary and classifies the population into the five SJL groups indicated in the legends. *p* values were calculated using the Pearson’s chi-square test (*p* = 0.379) and are comparative between the first trimester of the two phases of the study.

Figure 2 shows the distribution of SJL of all pregnant women in the first phase analysis (n = 205) and longitudinal phase (n = 100). The majority (54.5%) presented SJL higher than 1 h in the first trimester of pregnancy in the first phase, and 45% presented SJL higher than 1 h in the first trimester of pregnancy in the longitudinal phase.

**Figure 3:**
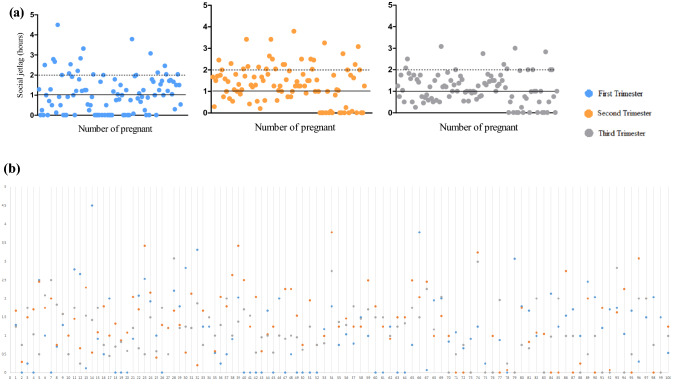
(**a**) Distribution of social jetlag (SJL) (hours) throughout each gestational trimester in the longitudinal phase. (b) The SJL value in hours is shown for each pregnant woman according to the three gestational trimesters (n = 100).

The distribution of SJL of the pregnant women of the longitudinal phase over the three gestational trimesters is presented in Fig. 3.

**Figure 4:**
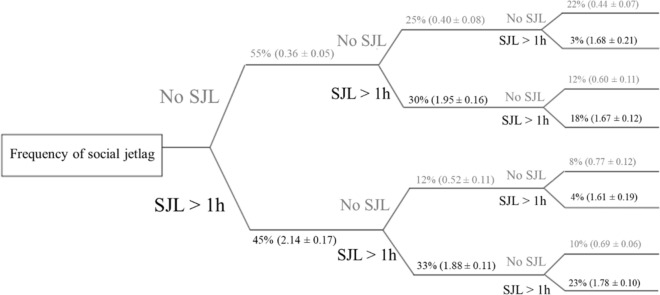
Trajectory of social jetlag (SJL) (No ≤ 1 h or > 1 h) category across three trimesters in the pregnant women in the longitudinal phase (n = 100). The categories were summarized using frequencies (%). Means ± standard error (SE) of each category are presented in parentheses.

Figure 4 shows the trajectory of the categories of SJL (> 1 h) over the three gestational trimesters in the longitudinal phase. A total of 77 (77.0%) pregnant women had no SJL higher than 1 h during at least one trimester, 22 (22.0%) had no SJL higher than 1 h in all three trimesters and 23 consistently had SJL higher than 1 h in all three trimesters (23.0%).

**Figure 5:**
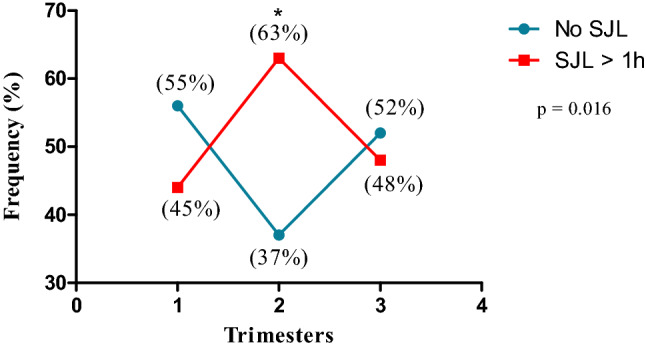
Frequency of social jetlag (SJL) by time category in pregnant participants over the three gestational trimesters in the longitudinal phase. The number of participants in each category is shown in parentheses (n = 100; n = 100 first trimester; 100 s trimester; and 100 third trimester). *Note*: *p* values were calculated using the Pearson’s chi-square test (*p* < 0.05) and are comparative between the trimester of the longitudinal phase.

Figure 5 presents the frequency of SJL higher than 1 h of pregnant women over the three gestational trimesters. The second gestational trimester showed the higher number of pregnant women with SJL higher than 1 h (63.0%) when compared to the first and third trimester (44.0% and 48.0%, respectively).

Table 2 presents the results of the linear regression that tested the associations between SJL and anthropometric variables. We found a positive association between SJL and pre-gestational BMI in the third trimester (β = 0.200, *p* = 0.046). No other significant associations were found.

**Table 2:** Linear regression analysis associating social jetlag (dependent variable) with anthropometric variables (independent variable) in the baseline (n = 205) and over the trimesters in the longitudinal phase (n = 100).

*Anthropometric variables*	Baseline		Longitudinal phase					
	1st trimester (n = 205)		1st trimester (n = 100)		2nd trimester (n = 100)		3rd trimester (n = 100)	
	β	*p* value	β	*p* value	β	*p* value	β	*p* value
Pre-gestational BMI (kg/m^2^)	0.130	0.083	0.072	0.479	0.115	0.254	0.200	**0.046**
Current BMI (kg/m^2^)	0.115	0.128	0.115	0.255	0.137	0.189	0.128	0.209
Weight gain (kg)	− 0.127	0.070	− 0.021	0.833	0.064	0.543	0.020	0.850

Linear regression analysis adjusted model for age, marital status, schooling, work (yes or no), gestational age and parity. Bold value is statistically significant at *p* < 0.05. *BMI* body mass index.

Table 3 presents the association between SJL and sleep patterns with anthropometric variables in the first phase, as well as throughout the trimesters. Analyses were adjusted for confounding variables. We did not find differences in SJL (dependent variable) between BMI categories (independent variable) in the baseline (pre-gestational: *p* = 0.071; current: *p* = 0.165). However, in the longitudinal phase, we found a significant effect of the interaction between gestation time (first, second and third trimester) and the categories of pre-gestational BMI (independent variables) on SJL (dependent variable). This indicates that normal weight pregnant women had decreased SJL from the second to the third trimester (1.29 ± 0.11 and 0.93 ± 0.08, respectively, *p* = 0.032), which did not happen with the three other groups of nutritional states. We also found an isolated significant effect of the gestation time on the mean of SJL; in this sense, pregnant women had decreased SJL from the second to the third trimester (1.33 ± 0.08 versus 1.12 ± 0.07, *p* = 0.012, respectively).

Regarding the sleep pattern, we found an isolated significant effect of the gestation time on the mean of week sleep time. In this sense, pregnant women had increased the week sleep time from the second to the third trimester (22:48 ± 0:08 versus 22:58 ± 0:07, *p* = 0.034, respectively). We also found differences in week awake time (dependent variable) between pre-gestational BMI categories (independent variable) in the longitudinal phase, indicating that underweight wake up later when compared to the overweight (8:14 ± 0:17 versus 7:01 ± 0:13, *p* = 0.039, respectively).

**Table 3:** Association of social jetlag and sleep patterns with anthropometric variables in the baseline (n = 205) and throughout the trimesters in the longitudinal phase (n = 100).

Variables	Baseline		Longitudinal phase					
	1st trimester (n = 205)		Overall (n = 100)		1st trimester (n = 100)	2nd trimester (n = 100)	3rd trimester (n = 100)	** *p* value
	(N) Mean ± SE or median [interquartile range]	*p value	Mean ± SE	** *p* value	Mean ± SE	Mean ± SE	Mean ± SE	
**Social jetlag**†	1.17 [0.00–5.50]		1.19 ± 0.88		1.10 ± 0.10^ab^	1.33 ± 0.08^a^	1.12 ± 0.07^b^	**0.012**
***Pre-gestational BMI****‡*								
Underweight	(18) 1.35 ± 0.28	0.071	1.17 ± 0.09	0.216	1.33 ± 0.28^ab^	1.04 ± 0.15^ab^	1.13 ± 0.23^ab^	**0.032**
Normal weight	(110) 1.27 ± 0.11		1.08 ± 0.08		1.02 ± 0.13^ab^	1.29 ± 0.11^a^	0.93 ± 0.08^b^	
Overweight	(42) 1.41 ± 0.16		1.30 ± 0.13		1.00 ± 0.19^ab^	1.48 ± 0.22^ab^	1.41 ± 0.14^ab^	
Obesity	(35) 1.94 ± 0.22		1.43 ± 0.15		1.50 ± 0.31^ab^	1.37 ± 0.19^ab^	1.41 ± 0.16^ab^	
***BMI—current****‡*								
Underweight	(20) 1.42 ± 0.22	0.165	1.15 ± 0.14	0.121	1.05 ± 0.27	1.40 ± 0.15	1.00 ± 0.20	0.478
Normal weight	(108) 1.22 ± 0.13		1.02 ± 0.09		1.06 ± 0.14	1.08 ± 0.13	0.93 ± 0.10	
Overweight	(50) 1.49 ± 0.16		1.30 ± 0.10		1.05 ± 0.18	1.64 ± 0.16	1.21 ± 0.13	
Obesity	(27) 1.77 ± 0.21		1.38 ± 0.16		1.37 ± 0.30	1.39 ± 0.22	1.38 ± 0.14	
**Week sleep time (h:min)¥**	22:51 ± 01:25		22:49 ± 1:08		22:48 ± 0:08^ab^	22:43 ± 0:05^a^	22:58 ± 0:07^b^	**0.034**
***Pre-gestational BMI****‡*								
Underweight	(18) 22:49 ± 0:20	0.308	22:40 ± 0:09	0.187	22:45 ± 0:19	22:30 ± 0:10	22:45 ± 0:06	0.091
Normal weight	(110) 22:57 ± 0:08		22:57 ± 0:08		22:52 ± 0:11	22:49 ± 0:08	23:10 ± 0:10	
Overweight	(42) 22:54 ± 0:12		22:45 ± 0:08		22:58 ± 0:12	22:37 ± 0:11	22:40 ± 0:11	
Obesity	(35) 22:23 ± 0:16		22:26 ± 0:11		22:11 ± 0:12	22:25 ± 0:12	22:44 ± 0:10	
***BMI—current****‡*								
Underweight	(20) 23:06 ± 0:16	0.180	23:20 ± 0:16	0.075	23:19 ± 0:23	23:14 ± 0:15	23:28 ± 0:17	0.011¢
Normal weight	(108) 22:48 ± 0:09		22:46 ± 0:09		22:40 ± 0:11	22:43 ± 0:09	22:53 ± 0:13	
Overweight	(50) 23:01 ± 0:11		22:50 ± 0:08		22:56 ± 0:14	22:33 ± 0:08	23:03 ± 0:10	
Obesity	(27) 22:23 ± 0:09		22:32 ± 0:09		22:28 ± 0:17	22:29 ± 0:10	22:42 ± 0:11	
**Weekend sleep time (h:min)¥**	23:24 ± 01:37		23:51 ± 1:07		23:52 ± 0:06	23:53 ± 0:06	23:49 ± 0:06	0.746
***Pre-gestational BMI****‡*								
Underweight	(18) 23:56 ± 0:47		23:55 ± 0:08		24:15 ± 0:13	23:25 ± 0:17	24:05 ± 0:14	
Normal weight	(110) 23:50 ± 0:19	0.878	23:52 ± 0:08	0.941	23:43 ± 0:09	24:00 ± 0:08	23:51 ± 0:10	0.088
Overweight	(42) 23:25 ± 0:26		23:53 ± 0:11		24:08 ± 0:13	23:50 ± 0:14	23:40 ± 0:14	
Obesity	(35) 23:47 ± 0:36		23:46 ± 0:11		23:48 ± 0:26	23:42 ± 0:10	23:48 ± 0:10	
***BMI—current****‡*								
Underweight	(20) 24:13 ± 0:36	0.861	24:15 ± 0:12	0.009¢	24:23 ± 0:13	24:06 ± 0:16	24:16 ± 0:17	0.017¢
Normal weight	(108) 23:40 ± 0:20		23:40 ± 0:10		23:35 ± 0:10	23:46 ± 0:11	23:37 ± 0:13	
Overweight	(50) 23:37 ± 0:25		24:06 ± 0:07		24:01 ± 0:16	24:16 ± 0:08	24:03 ± 0:10	
Obesity	(27) 23:58 ± 0:34		23:40 ± 0:09		24:01 ± 0:14	23:24 ± 0:12	23:34 ± 0:13	
**Week awake time (h:min)¥**	7:20 ± 01:30		7:24 ± 1:31		7:31 ± 0:10	7:17 ± 0:09	7:23 ± 0:08	0.464
***Pre-gestational BMI****‡*								
Underweight	(18) 8:09 ± 0:26	0.068	8:14 ± 0:17^a^	**0.039**	8:15 ± 0:47	8:20 ± 0:22	8:08 ± 0:22	0.223
Normal weight	(110) 7:31 ± 0:10		7:30 ± 0:10^ab^		7:31 ± 0:12	7:26 ± 0:11	7:37 ± 0:10	
Overweight	(42) 7:22 ± 0:13		7:01 ± 0:13^b^		7:35 ± 0:21	6:42 ± 0:20	6:49 ± 0:14	
Obesity	(35) 6:52 ± 0:17		7:13 ± 0:20^ab^		7:04 ± 0:28	7:17 ± 0:23	7:16 ± 0:21	
***BMI—current****‡*								
Underweight	(20) 9:34 ± 0:19	0.102	8:02 ± 0:23	0.186	8:17 ± 0:37	7:55 ± 0:23	7:56 ± 0:22	0.521
Normal weight	(108) 8:50 ± 0:10		7:26 ± 0:10		7:26 ± 0:10	7:26 ± 0:14	7:26 ± 0:13	
Overweight	(50) 9:07 ± 0:13		7:09 ± 0:11		7:16 ± 0:17	6:52 ± 0:15	7:21 ± 0:13	
Obesity	(27) 8:37 ± 0:17		7:14 ± 0:18		7:31 ± 0:37	7:10 ± 0:26	7:02 ± 0:19	
**Weekend awake time (h:min)¥**	8:37 ± 01:23		8:46 ± 1:25		8:48 ± 0:08	8:55 ± 0:09	8:37 ± 0:07	0.125
***Pre-gestational BMI****‡*								
Underweight	(18) 9:44 ± 0:25	0.171	9:19 ± 0:08	0.020¢	10:10 ± 0:35	8:45 ± 0:17	9:05 ± 0:16	0.067
Normal weight	(110) 8:54 ± 0:09		8:45 ± 0:10		8:39 ± 0:10	9:00 ± 0:13	8:36 ± 0:10	
Overweight	(42) 9:08 ± 0:14		8:45 ± 0:11		8:59 ± 0:18	8:48 ± 0:18	8:28 ± 0:13	
Obesity	(35) 8:39 ± 0:17		8:41 ± 0:14		8:30 ± 0:22	8:51 ± 0:17	8:44 ± 0:19	
***BMI—current****‡*								
Underweight	(20) 9:34 ± 0:19	0.102	9:16 ± 0:17	0.282	9:44 ± 0:23	8:50 ± 0:19	9:16 ± 0:28	0.269
Normal weight	(108) 8:50 ± 0:10		8:39 ± 0:10		8:34 ± 0:13	8:54 ± 0:17	8:30 ± 0:13	
Overweight	(50) 9:07 ± 0:13		8:41 ± 0:09		8:47 ± 0:14	8:56 ± 0:13	8:22 ± 0:13	
Obesity	(27) 8:37 ± 0:17		8:52 ± 0:15		8:45 ± 0:24	9:00 ± 0:21	8:51 ± 0:13	

*SE* standard error; *BMI* body mass index. *p values calculated by—generalised linear models (GzLM) (mean ± standard deviation) in the baseline. Adjusted to age, marital status, schooling, work (yes or no), gestational age and parity. Sidak post-hoc test, *p* value < 0.05 was considered significant. **p values calculated by Generalized Estimating Equation (GEE) (mean ± standard deviation) in the longitudinal phase. Significant results of the models were shown in bold. †Adjusted for age, marital status, schooling, work (yes or no), parity, body mass index and Pittsburgh Sleep Quality Index Global Sleep Quality Score. ‡Adjusted to age, marital status, schooling, work (yes or no), parity and gestational age. **¥**Time is presented in 24-h clock time. Sidak post-hoc test, *p* value < 0.05 was considered significant. ¢The sequential Sidak’s post-test did not identify any differences between the BMI categories. Total gestational data’s values represent the average of the three trimesters.

Table 4 shows the odds ratio (OR) for having > 1 h of SJL according to adequacy of pre-gestational BMI, current BMI and weight gain in each gestational trimester. Results indicated a higher risk of having SJL higher than 1 h in the third trimester when pregnant women had inadequate pre-gestational BMI (OR = 3.059, IC 95% = 1.343–6.964), and also in the second trimester when pregnant women had inadequate current BMI (OR = 3.470, IC 95% = 1.490–8.081).

**Table 4:** Odds ratio (OR) for having > 1 h of social jetlag according to anthropometric variables categories in the gestational trimesters (reference group: ≤ 1 h of social jetlag).

	Baseline				Longitudinal phase			
	1st trimester		1st trimester		2nd trimester		3rd trimester	
	OR (IC 95%)	*p* value	OR (IC 95%)	*p* value	OR (IC 95%)	*p* value	OR (IC 95%)	*p* value
Pre-gestational BMI								
**Adequate** (reference group)	1.0	0.938	1.00	0.898	1.00	0.577	1.00	**0.008**
**Inadequate**	1.024 (0.568–1.847)		1.113 (0.502–2.466)		1.263 (0.556–2.869)		3.059 (1.343–6.964)	
Current BMI								
**Adequate** (reference group)	1.00	1.000	1.00	0.904	1.00	**0.004**	1.00	0.182
**Inadequate**	1.000 (0.550–1.820)		0.952 (0.432–2.099)		3.470 (1.490–8.081)		1.769 (0.765–4.091)	
Weight gain								
**Adequate** (reference group)	1.00	0.431	1.00	0.389	1.00	0.361	1.00	0.389
**Inadequate**	1.554 (0.97–4.87)		0.683 (0.287–1.626)		0.652 (0.260–1.632)		0.683 (0.287–1.626)	

Logistic regressions analysis adjusted model for age, marital status, schooling, work (yes or no), parity and gestational age. Bold value is statistically significant at *p* < 0.05. *IC* confidence interval.

**Figure 6:**
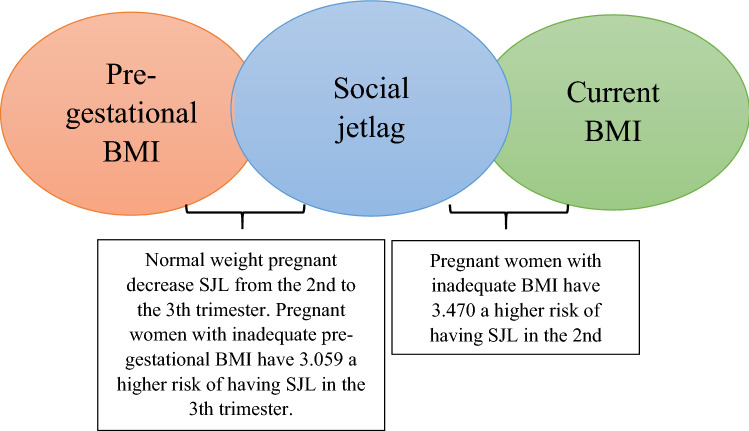
Relationship between social jetlag, pre-gestational BMI and current BMI.

Figure 6 illustrates the possible effect of pre-gestational and current BMI on the SJL according to the previously analyses presented. We found a relationship between pre-pregnancy BMI and SJL, in which pregnant women classified as normal weight in the pre-gestational BMI decreased the SJL from the second to the third trimester (Table 3). A relationship between the current BMI and SJL was also observed, in which pregnant women classified as inadequate in current BMI (underweight, overweight and obesity) presented a higher chance of having SJL higher than 1 h when compared to those with normal weight (Table 4).

## Discussion

The present study aimed to identify the prevalence of SJL and to to verify whether there is an effect of pre-pregnancy BMI on SJL throughout pregnancy. To the best of our knowledge, this is the first cohort study to describe SJL over the gestational period. Our results indicated a high prevalence of circadian misalignment measured by SJL, with most of the pregnant women (54.5%) presenting SJL higher than 1 h in the first gestational trimester and 77.0% of the pregnant women having SJL higher than 1 h during at least one trimester. In the longitudinal phase, we found that normal BMI pregnant women tend to have decreased SJL from the second to the third trimester, which was not found in underweight, overweight and obese pregnant women. Our results also indicated a higher risk of having SJL higher than 1 h in the second and third trimester when pregnant women had inadequate BMI. These results partially confirm our hypothesis that SJL changes throughout the gestational period. Regarding our initial hypothesis that relates the occurrence of SJL higher than 1 h to body weight status, we found that pregnant women with excessive weight tend to maintain the degree of pre-gestational circadian misalignment, while pregnant women with normal weight decrease SJL over the gestation.

Studies in animal models have demonstrated that genetic disruption of the circadian clock negatively influences pregnancy outcomes^25,26,38,39^. The effects of circadian system environmentally-imposed disturbances on mammalian model pregnancy seems to dramatically reduce the pregnancy success^27^, decrease the number of births^28^ and disturbed the fetal intrauterine growth and the growth of neonatal rats^28^. Epidemiological studies have also showed an association between gestational chronodisruption—as usual in shift work^40–42^, travel across time zones^43,44^, or light exposure at night^45^—and adverse pregnancy outcomes in humans, as fetal loss and spontaneous abortion^46^, duration of pregnancy (including preterm or postterm birth)^46,47^ and low birth weight and/or small for gestational age^48,49^. Also, studies suggest that pregnant night workers might have an increased risk of miscarriage^45^, hypertensive disorders during pregnancy^50^ and preterm delivery^51^. Thus, the high prevalence of SJL higher than 1 h in pregnant women should be considered very worrying.

Unfortunately, the prevalence of SJL in pregnant women is currently unknown, which makes it impossible to compare our findings with previous studies. However, studies conducted with other groups have found that SJL has been highly prevalent among young people, adults and the elderly^8,9,52^. Two studies developed with Germany population found a SJL prevalence among 69–70%^8,52^. Roenneberg et al. (2012)^9^, in an epidemiologic study developed with 65,000 European participants, found that 33% of them have SJL ≥ 2 h and 69% have, at least, 1 h of SJL. Also, a Netherlander study (n = 1585, 60.8 ± 6y ) found that 31% of the sample reported SJL between 1 and 2 h and 8% reported SJL > 2 h^11^. In addition, Lang et al. (2018)^53^ found that 31.1% of Australian people have SJL > 1 h (n = 837; 18–75y). In Brazil, studies from our group with shift workers^54^, university students^55,56^ and patients with chronic diseases^16,17^ have followed the same pattern as in the other countries previously mentioned.

Previous studies have shown that age variation is among the factors that can influence the SJL^9,10,14^. This can explain the high SJL prevalence found in the present study since our sample is young. Another factor that is frequently associated with SJL is chronotype. This variability in the sleep–wake cycle is especially evident among late types, who generally report a greater accumulated sleep debt during the week (Roenneberg et al., 2012)^9^. Taillard et al. (1999)^57^ found that around 72.5% of evening types changed their bedtime and wake up time in, at least, two hours between the workdays and the weekend, compared to 49.8% of morning types (circadian preference defined by Horne and Ostberg, 1976^58^). In fact, individuals with eveningness tendency are more susceptible to SJL than those with morningness tendency^8,59–61^, since this sleep–wake cycle irregularity during the week and on weekend was considered a compensatory strategy for the evening types, who are generally more susceptible to asynchrony in their ''biological'' and ''social'' clocks^62^.

Our results also show that pregnant women had decreased SJL from the second to the third trimester. Such results demonstrate that the degree of SJL tends to change during pregnancy, which can be justified by the fact that each gestational trimester have specific characteristics that could influence the sleep quality. There is, for example, an increase in sleep duration, daytime sleepiness and insomnia in the first trimester of pregnancy, while sleep quality tends to decrease^63,64^ due to nocturnal urinary frequency and heartburn^65^. In the second trimester, in addition to the factors already mentioned, fetal movement and frequent heartburn can disturb sleep^66^. Lastly, in the third trimester sleep pattern can be altered due to abdominal increase and anxiety^67–69^. As many pregnant women in our sample (57%) weren't working in the third trimester, this may have reduced social demand and minimized the effects of sleep problems, impacting on the SJL in this trimester. However, studies in the literature that assessed SJL in a longitudinal context are still limited^61,70^ and have not been conducted with pregnant women, which makes it impossible to compare with our findings.

SJL has been identified as a possible risk factor for the development of excess weight in non-pregnant populations^9,10,16,71^. It is still possible that there is a bidirectional relationship between SJL and body weight, given that excess weight also seems to predispose one to an increase in SJL^72^, as found by a Chinese population study that found that BMI seems to be a positive predictor for SJL (*p* = 0.017)^72^. This result corroborates ours, which indicates a higher risk of having SJL higher than 1 h in the second and third gestational trimester when pregnant women had inadequate BMI. Although the mechanisms are not completely elucidated, humoral, genetic, feeding habits and behavioral factors are identified as possibly responsible for the association between excess weight and the development of circadian misalignment^73^. Additional studies are needed to understand the causality relationship between body weight and SJL.

As a strong point of our study, we highlight the SJL analysis at the beginning and also during pregnancy, which allowed us to evaluate SJL changes over time. We emphasize as a limitation, the use of subjective questionnaires, which, although validated in other studies, are dependent on the memory and motivation of the participants. Another limitation in our study is that we followed-up pregnant women who had regular appointments in the public health system, and the generalization of the results for all pregnant women cannot be done, especially in high-risk pregnant women. Also, the selection of pregnant women who were followed throughout the pregnancy was not random, which may have led to selection bias. Despite these limitations, we expect that the results of the present study can improve the understanding of the association between SJL and anthropometrics variables during pregnancy. However, the need for further studies on this subject is evident.

We conclude that SJL is quite prevalent during the gestational period and excessive BMI both before and during pregnancy is associated with an increased risk of having SJL > 1 h in the third and second trimesters, respectively. In addition, pregnant women of normal weight—but not underweight or overweight—had decreased SJL from the second to the third trimester. Thus, new studies that include these variables may lead to a better understanding of the dynamics of SJL and the factors that contribute to increased SJL risk may be important. If our findings are confirmed in future studies, monitoring chronobiological variables such as SJL in promoting maternal and fetal health may emerge as a strategy to improve the effectiveness of prenatal care.
